# Sub-Inhibitory Concentrations of Trans-Cinnamaldehyde Attenuate Virulence in *Cronobacter sakazakii in Vitro*

**DOI:** 10.3390/ijms15058639

**Published:** 2014-05-15

**Authors:** Mary Anne Roshni Amalaradjou, Kwang Sik Kim, Kumar Venkitanarayanan

**Affiliations:** 1 Department of Animal Science, University of Connecticut, 3636 Horse Barn Hill Road Ext., Unit 4040, Storrs, CT 06269, USA; E-Mail: mary_anne.amalaradjou@uconn.edu; 2 Division of Pediatric Infectious Diseases, School of Medicine, Johns Hopkins University, Baltimore, MD 21218, USA; E-Mail: kwangkim@jhmi.edu

**Keywords:** *C. sakazakii*, trans-cinnamaldehyde, sub-inhibitory concentration, virulence, attenuation

## Abstract

*Cronobacter sakazakii* is a foodborne pathogen, which causes a life-threatening form of meningitis, necrotizing colitis and meningoencephalitis in neonates and children. Epidemiological studies implicate dried infant formula as the principal source of *C. sakazakii*. In this study, we investigated the efficacy of sub-inhibitory concentrations (SIC) of trans-cinnamaldehyde (TC), an ingredient in cinnamon, for reducing *C. sakazakii* virulence *in vitro* using cell culture, microscopy and gene expression assays. TC significantly (*p* ≤ 0.05) suppressed *C. sakazakii* adhesion to and invasion of human and rat intestinal epithelial cells, and human brain microvascular endothelial cells. In addition, TC inhibited *C. sakazakii* survival and replication in human macrophages. We also observed that TC reduced the ability of *C. sakazakii* to cause cell death in rat intestinal cells, by inhibiting nitric oxide production. Results from gene expression studies revealed that TC significantly downregulated the virulence genes critical for motility, host tissue adhesion and invasion, macrophage survival, and LPS (Lipopolysaccharide) synthesis in *C. sakazakii*. The efficacy of TC in attenuating these major virulence factors in *C. sakazakii* underscores its potential use in the prevention and/or control of infection caused by this pathogen.

## 1. Introduction

*Cronobacter sakazakii* is a food-borne pathogen associated with severe illness and high mortality in neonates and infants [1,2]. Infection with *C. sakazakii* (CS) results in bacteremia, sepsis, meningitis, and brain abscess or cyst formation [3]. *C. sakazakii* is also associated with necrotizing enterocolitis (NEC), characterized by bacterial colonization of the gastrointestinal lumen [4]. The infant mortality rate associated with *C. sakazakii* infections ranges from 40% to 80% [5], and up to 20% of infected newborns develop severe neurological sequelae such as hydrocephalus, quadriplegia and retarded neural development [6].

The primary means of contracting *C. sakazakii* infection is oral, since feeding of contaminated powdered infant formula has been established as the only known link to infection in neonates [4,7]. Therefore, the most common colonization site of this pathogen is the intestinal tract, and presence of *C. sakazakii* in stool samples without overt signs of infection has been reported [8]. Thus, attachment and invasion of intestinal epithelial cells by *C. sakazakii* is the first critical step in establishing a successful systemic infection. Mange and others [9] demonstrated the ability of *C. sakazakii* to adhere and invade human epithelial and brain microvascular endothelial cells. Our laboratory previously reported that outer membrane protein A (OmpA) in *C. sakazaki* binds to fibronectin, and plays an important role in the invasion of human intestinal and brain microvascular endothelial cells [10,11]. In addition to its ability to invade different cell types, *C. sakazakii* is capable of surviving and replicating within macrophages. The ability of *C. sakazakii* to survive, replicate and thrive in these abundant immune cells has been suggested as an advantageous component of its pathogenic potential [12].

Trans-cinnamaldehyde (TC) is a major component of bark extract of cinnamon. It is classified as a generally regarded as safe (GRAS) molecule by the FDA (Food and Drug administration) [13]. Our laboratory previously reported that TC was effective in inhibiting *C. sakazakii* biofilm synthesis by down-regulating several biofilm-associated genes in the pathogen [14]. In a follow up study using proteomics, we observed that TC exerted antimicrobial effects on *C. sakazakii* by multiple mechanisms, including interference with motility, invasion ability, and cellular defenses of the pathogen against oxidative stress [15]. In this study, we demonstrate the efficacy of sub-inhibitory concentrations (SIC, concentrations not inhibiting growth) of TC for reducing *C. sakazakii* virulence *in vitro*. Additionally, the effect of TC on the expression of major virulence genes in *C. sakazakii* was determined.

## 2. Results and Discussion

### 2.1. Results

#### 2.1.1. Sub-Inhibitory Concentrations of TC (Trans-Cinnamaldehyde)

The three SICs of TC that allowed *C. sakazakii* growth similar to control (0 μM TC) were 325, 560 and 750 μM. The average initial *C. sakazakii* population in the control and TC-treated samples was approximately 6.2 log CFU/mL. Following incubation at 37 °C for 24 h, approximately 8.0 log CFU/mL of *C. sakazakii* was recovered from all wells, irrespective of control and TC treatment (data not shown). This confirmed that the TC concentrations used in the assay (325, 560 and 750 μM) were not inhibitory to the bacteria. Since no significant differences (*p* > 0.05) were observed for any of the tested parameters between strains or tissue culture types, the results obtained from CS (*C. sakazakii*) ATCC 29544 strain and IEC (Intestinal Epithelial Cells)-6 cells are provided in the manuscript, unless mentioned otherwise.

#### 2.1.2. TC Suppresses *C. sakazakii* Motility

**Figure 1.**
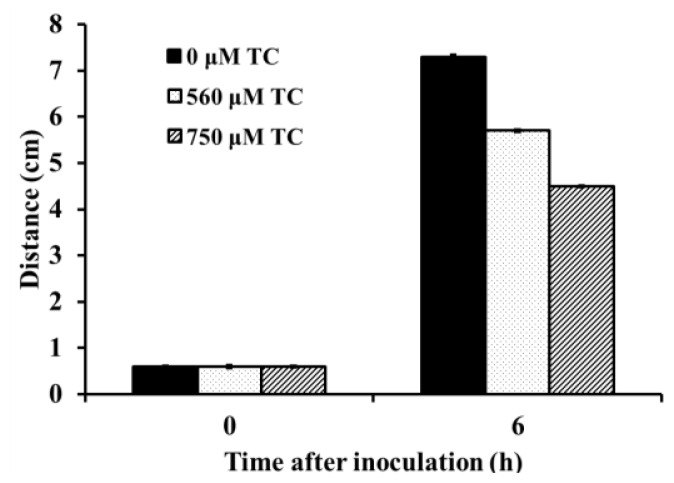
Effect of sub-inhibitory concentrations (SIC) of TC (Trans-Cinnamaldehyde) on *C. sakazakii* ATCC 29544 motility. Petriplates containing 20 mL of LB broth + 0.3% agar at 45 °C were inoculated with *C. sakazakii* culture (grown in the presence of 0, 560 or 750 μM TC) containing ~6.0 log CFU and the plates were kept still for 1 h at room temperature, followed by incubation upside down at 37 °C for 7 h.

The effect of SIC’s of TC on *C. sakazakii* ATCC 29544 motility is shown in Figure 1. In control samples, swarming *C. sakazakii* were able to reach the edge of the plate 7 h after inoculation (7.3 mm motility zone diameter). However, in the plates inoculated with *C. sakazakii* treated with 560 and 750 μM of TC, the diameter of the motility zone was significantly reduced (*p* < 0.05). TC was found to be equally effective against all the three *Cronobacter sakazakii* isolates (ATCC 29544, ATCC 29004 and CS 415). Therefore, only the results obtained with CS ATCC 29544 are presented here.

#### 2.1.3. TC Suppresses *C. sakazakii* Adhesion and Invasion of Host Cells

**Figure 2.**
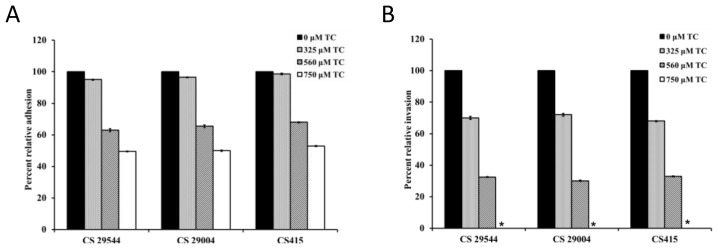
Effect of TC on adhesion (**A**) and invasion (**B**) of IEC (Intestinal Epithelial Cells)-6 cells by *C. sakazakii*. IEC-6 cells were inoculated with *C. sakazakii* grown to midlog phase with SICs of TC (0, 325, 560 and 750 μM) at a MOI (Multiplicity of Infection) of 10. Following inoculation, the tissue culture trays were incubated at 37 °C in a humidified, 5% CO_2_ incubator. The infected monolayers were rinsed in PBS (phosphate-buffered saline) after 1 h of incubation, and the cells were lysed with 0.1% Triton X-100. The number of viable adherent *C. sakazakii* was determined by plating. For the internalization assay, the monolayers were incubated for 1 h following infection, rinsed in minimal media and incubated for another 2 h in whole media-1% FBS (fetal bovine serum) containing gentamicin (100 μg/mL) to kill the extracellular bacteria. The invaded bacteria were enumerated following serial dilution and plating. * No invaded bacteria recovered from 750 μM TC treatment.

**Figure 3.**
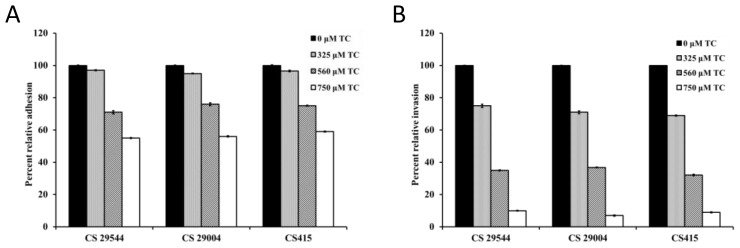
Effect of TC on adhesion (**A**) and invasion (**B**) of BMEC cells by *C. sakazakii*. BMEC cells were inoculated with *C. sakazakii* grown to midlog phase with SICs of TC (0, 325, 560 and 750 μM) at a MOI of 10. Following inoculation, the tissue culture trays were incubated at 37 °C in a humidified, 5% CO_2_ incubator. The infected monolayers were rinsed three times in PBS after 1 h of incubation, and the cells were lysed with 0.1% Triton X-100. The number of viable adherent *C. sakazakii* was determined by plating. For the internalization assay, the monolayers were incubated for 1 h following infection, rinsed in minimal media and incubated for another 2 h in whole media-1% FBS containing gentamicin (100 μg/mL) to kill the extracellular bacteria. The invaded bacteria were enumerated following serial dilution and plating.

**Figure 4.**
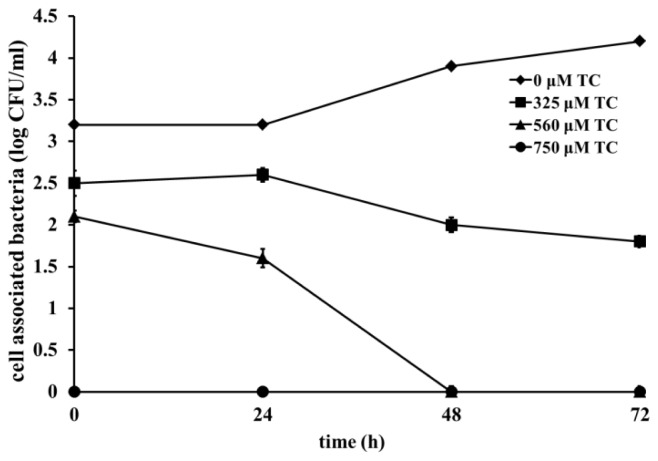
Effect of TC on intracellular survival and replication of *C. sakazakii* ATCC 29544 within macrophages. U937 cells were inoculated with *C. sakazakii* grown to midlog phase with SICs of TC (0, 325, 560 and 750 μM) at an MOI of 10. Following inoculation, the tissue culture trays were incubated for 45 min at 37 °C under 5% CO_2_. After incubation, the macrophages were resuspended in U937 medium supplemented with 100 μg of gentamicin/mL and were incubated for an additional 45 min at 37 °C under 5% CO_2_. Macrophages were then lysed with 0.5% Triton X, serially diluted, and plated. For extended assays (intracellular replication assays), the cells were replenished with fresh medium containing 10 μg of gentamicin/mL. For each indicated time point, results are presented as percent survival of the initial intracellular population recovered at time zero.

The effect of TC on the adhesion and invasion of *C. sakazakii* strains in IEC-6 cells is depicted in Figure 2. Following the adhesion assay, it was observed that 560 and 750 μM of TC inhibited adhesion (*p* < 0.05) of CS 2 by ~35% and 47%, respectively, compared to control (Figure 2A). Similar results were obtained with the other three strains tested. TC was also effective in inhibiting (*p* < 0.05) the ability of *C. sakazakii* to invade IEC-6 cells. The invasiveness of CS ATCC 29544 was reduced by ~25% and 55%, by 325 and 560 μM of TC, respectively. Notably, the highest SIC of TC (750 μM) completely inhibited the invasion of IEC-6 cells by all four *C. sakazakii* strains (Figure 2B). Similar results were obtained with the adhesion and invasion studies performed using Caco-2 and INT-407 monolayers (data not shown). As observed with intestinal epithelial cells, TC was effective in inhibiting adhesion and invasion of BMEC cells by *C. sakazakii* (Figure 3A,B). Trans-cinnamaldehyde at 750 μM reduced *C. sakazakii* adhesion and invasion of BMEC cells by ~50% and 90%, respectively, compared to control. *C. sakazakii* was able to survive and replicate within human macrophages, as demonstrated by the increase in its population in TC-untreated samples (Figure 4). However, all three SICs of TC were able to significantly decrease replication of *C. sakazakii* in the macrophages. TC was found to be equally effective against all the three isolates of CS tested (ATCC 29544, ATCC 29004 and CS 415). No significant differences were observed between the different isolates (*p* < 0.05) in the macrophage assay.

#### 2.1.4. TC Suppresses Cell Death in Rat Intestinal Cells

**Figure 5.**
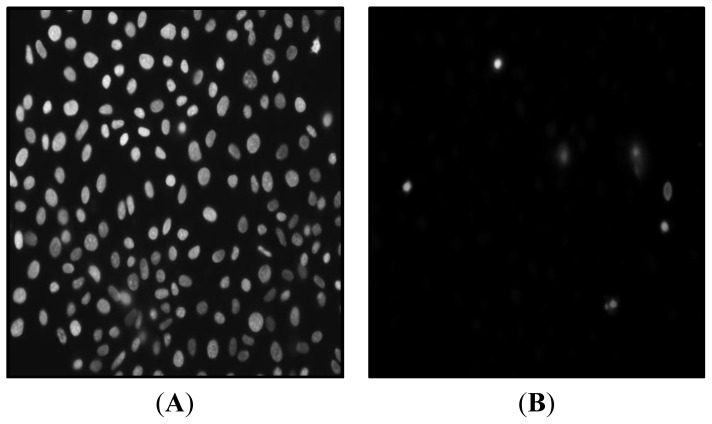
Fluorescent microscopy of IEC-6 cells infected with *C. sakazakii* ATCC 29544 untreated with TC (**A**) and treated with TC (**B**). Confluent IEC monolayers were infected with 100 MOI of *C. sakazakii* (grown to mid log phase in presence of TC at 0 and 750 μM) and incubated for 6 h at 37 °C under 5% CO_2_. Following incubation, the cells were washed and stained with DAPI (4-,6-diamidino-2-phenylindole) staining to visualize apoptosis and cell death using a fluorescence microscope.

The efficacy of TC to attenuate virulence of *C. sakazakii* by preventing cell death was investigated using DAPI (4-,6-diamidino-2-phenylindole) staining and fluorescence microscopy. The image of IEC-6 cells infected with *C. sakazakii* not treated with TC (Figure 5A) revealed the presence of brightly stained nuclei with deformations, as associated with dead or dying cells. However, the rat intestinal cells infected with TC-treated *C. sakazakii* appeared healthy and the nuclei did not take up DAPI, unlike the dead cells (Figure 5B). Additionally, no significant differences were observed between the different isolates (*p* < 0.05). Therefore, only the results obtained with CS ATCC 29544 are presented here.

**Figure 6.**
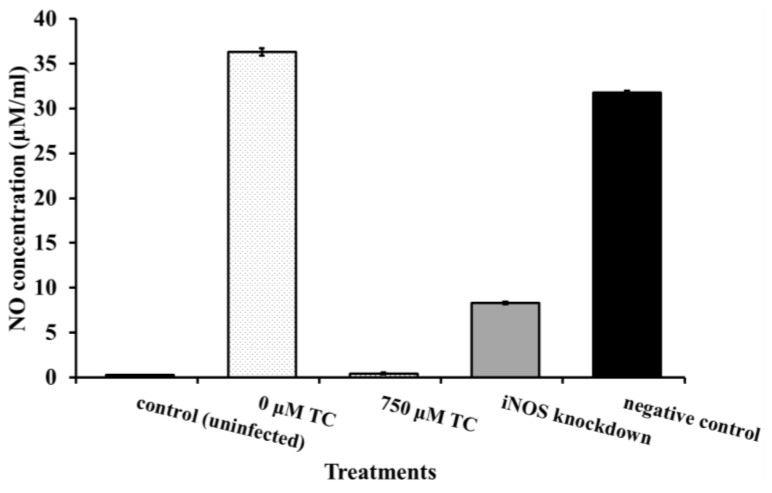
Effect of TC on NO (Nitric Oxide) production by IEC-6 cells challenged with *C. sakazakii* ATCC 29544. IEC-6 cells were grown to 50% confluencey in 6 well tissue culture plates and transfected with siRNA for iNOS (inducible nitric oxide synthase). Following transfection, *C. sakazakii* (grown to mid log phase in the presence of TC at 0 and 750 μM) was added to each well at a MOI of 100 and incubated for 4 h. The supernatants were subjected to centrifugation and used to estimate NO production.

**Figure 7.**
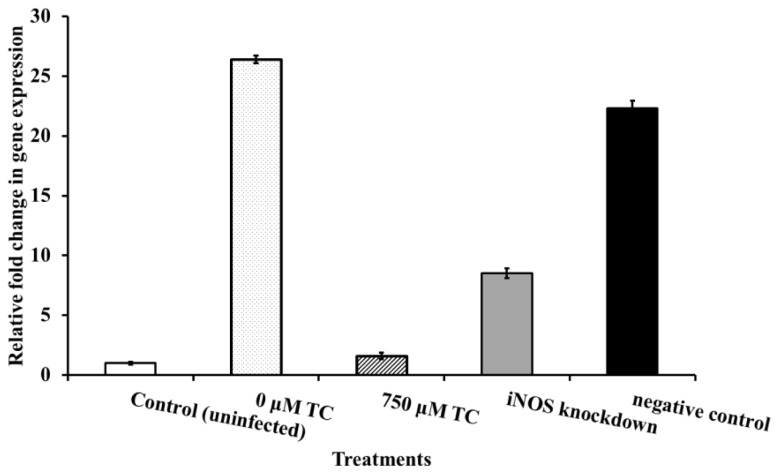
Effect of TC on the expression of iNOS gene by IEC-6 cells challenged with *C. sakazakii* ATCC 29544. IEC-6 cells were grown to 50% confluency in six well tissue culture plates and transfected with siRNA for iNOS. Following transfection, *C. sakazakii* (grown to mid log phase in the presence of TC at 0 and 750 μM) was added to each well at a MOI of 100 and incubated for 4 h. Total RNA was extracted from control (uninfected), *C. sakazakii*-infected and TC-treated-IEC-6 cells. Real-time qPCR for iNOS expression was performed using ribosomal protein S-17 (RPS-17) as an internal control.

Hunter *et al.* [16] demonstrated that necrotizing enterocolitis in rat intestinal cells caused by *C. sakazakii* can be attributed to the induction of nitric oxide production by the pathogen. To explain the reduced cell death observed in DAPI images of IEC-6 cells infected with TC-treated *C. sakazaki*i, we investigated the ability of TC to inhibit NO (Nitric Oxide) production following transfection of IEC-6 cells with iNOS (inducible nitric oxide synthase) siRNA. It was found that NO production in *C. sakazakii* infected IEC cells was 1700 μM, while that in the uninfected cells was 200 μM. In the rat intestinal cells that were infected with TC-treated *C. sakazakii*, 300 μM of NO was detected, which was comparable to that in uninfected, healthy cells (Figure 6). Gene expression studies revealed that transfection of IEC-6 cells with iNOS siRNA (positive control) resulted in a 75% knockdown of iNOS gene expression. However, iNOS expression in *C. sakazakii* infected IEC-6 cells was upregulated significantly compared to uninfected cells and cells transfected with siRNA. Notably, in the IEC cells inoculated with TC-treated *C. sakazakii*, iNOS gene expression was similar to that in the uninfected, healthy cells (Figure 7). Similar results were obtained will all the three CS isolates. Therefore, only the results obtained with CS ATCC 29544 are presented here.

#### 2.1.5. TC Reduces Endotoxin Production in *C. sakazakii*

**Figure 8.**
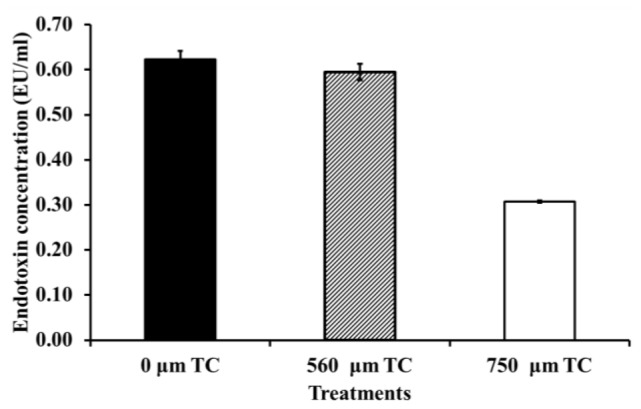
Effect of TC on endotoxin production by *C. sakazakii* ATCC 29544. *C. sakazakii* grown in TSB (tryptic soy broth) to midlog phase in the presence of SICs of TC (0, 560 and 750 μM) was analyzed for endotoxin production. Samples were analyzed in a 96-well format in a Bio-Rad microplate reader (Bio-Rad Laboratories, Hercules, CA, USA) using a ToxinSensor Chromogenic LAL Endotoxin Assay Kit (GenScript, Piscataway, NJ, USA).

Since endotoxin contributes to the virulence of Gram-negative bacteria, the ability of TC to inhibit endotoxin production in *C. sakazakii* was investigated. Although 560 μM of the plant molecule demonstrated a negligible inhibitory effect on endotoxin production, 750 μM TC decreased endotoxin synthesis by 50% (*p* < 0.05) in comparison to that in TC-untreated *C. sakazakii* (Figure 8). TC was found to be equally effective against all the three CS isolates (ATCC 29544, ATCC 29004 and CS 415). Therefore, only the results obtained with CS ATCC 29544 are presented here.

#### 2.1.6. TC Downregulates *C. sakazakii* Virulence Genes

**Figure 9.**
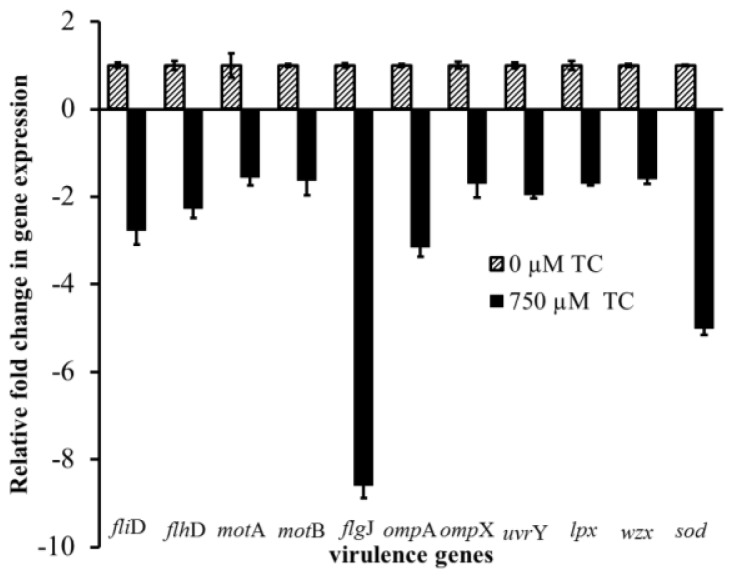
Effect of TC on expression of virulence genes in *C. sakazakii* ATCC 29544. Relative gene expression was assayed using the ABI Prism 7500 Fast Real Time PCR System. The data were normalized to the endogenous control (16s RNA) and the level of candidate gene expression between TC-treated and untreated samples was compared to study relative gene expression, and the effect of TC on tested genes.

To elucidate if the observed attenuating effect of TC on *C. sakazakii* virulence factors was due to its effect at the transcriptional level, RT-qPCR (real time-quantitative PCR) was performed on *C. sakazakii* RNA, using primers specific for the respective virulence genes. Results from RT-qPCR revealed that TC at 560 and 750 μM down-regulated (*p* < 0.05) the expression of all eleven *C. sakazakii* virulence genes assayed (Figure 9). It was observed that 750 μM of TC was more effective than 560 μM in inhibiting the expression of virulence genes. Among the various virulence genes studied, the expression of genes encoding *C. sakazakii* flagellar muramidase, superoxide dismutase and outer membrane protein A were more significantly down-regulated than others. No significant differences were observed between the different isolates (*p* < 0.05). Therefore, only the results obtained with CS ATCC 29544 are presented here.

### 2.2. Discussion

Although *C. sakazakii* is an opportunistic pathogen, it causes a life-threatening infection in neonates and infants, especially pre-term infants, with severe neurological sequelae in survivors [3]. Necrotizing enterocolitis caused by *C. sakazakii* is characterized by high morbidity and mortality, and few effective measures are available to prevent the disease [17]. The current treatment strategy against *C. sakazakii* infections includes administration of broad-spectrum antibiotics, but emerging resistance to antibiotics in *C. sakazakii* [18–20] underscores the need for alternate approaches to control this pathogen.

Sub-inhibitory concentrations of antimicrobials, including antibiotics are reported to modulate transcription of bacterial genes [21], and hence used to study the molecular mechanisms behind their antimicrobial effects [22]. In order to cause NEC, *C. sakazakii* must attach and invade the intestinal epithelial cells, thereby leading to host colonization. Further, to cause extra-intestinal infections such as sepsis and meningitis, the pathogen needs to break through the intestinal mucosa, gain access to the bloodstream, and survive the host defense mechanisms, before gaining entry into the central nervous system. Therefore, we investigated the effect of SICs of TC on adherence and invasion of *C. sakazakii* in cultured human intestinal epithelial cells, macrophages and brain cells.

The results from the cell culture studies revealed that TC considerably (*p* < 0.05) reduced the adhesive and invasive abilities of *C. sakazakii* in all three intestinal epithelial cell lines tested (Figure 2). As observed with the intestinal cells, TC was effective in markedly reducing the attachment and invasion of BMEC by *C. sakazakii* (Figure 3). The effect of TC was found to be concentration-dependent, with the highest SIC of 750 μM TC being more effective than the lower concentrations in decreasing the attachment and invasion of *C. sakazakii* in the cells. It was also observed that for any given SIC, TC was more effective in reducing the pathogen invasion than attachment to the cells. The reduced adhesive and invasive abilities of *C. sakazakii* observed for all the cells tested were supported by results from the gene expression studies. RT-qPCR results revealed that TC significantly downregulated *uvrY*, *ompA* and *ompX*, which have been shown to play a vital role in bacterial attachment and invasion of host tissue. For example, *uvrY* is part of the BarA-UvrY two component system that regulates virulence in *E. coli* O78:K80:H9 and *uvrY* mutants were compromised in their ability to adhere, invade and persist within host tissues [23]. Similarly, *ompA* is critical for *C. sakazakii* attachment and invasion of human intestinal epithelial cells [10,24] and BMEC [11,25]. *ompX* is yet another gene in *C. sakazakii* which was reported to play an important role in bacterial attachment and invasion of host cells and virulence in the host [24].

Bacterial motility is a complex network of multiple events, including signal transduction, chemotaxis and flagellar movement [26,27]. Motility plays a critical role in host-microbial interactions, bacterial colonization and virulence in the host [27,28]. Flagellar and non-flagellar mediated motility aid invasion and colonization of *S. enteritidis* [28] and *C. jejuni* [29] *in vitro* and *in vivo*. Therefore, the effect of TC on *C. sakazakii* motility was investigated. The results from the motility assay revealed that TC significantly reduced motility in *C. sakazakii* (Figure 1). RT-qPCR results confirmed that TC downregulated the genes associated with the flagellar apparatus and motility in *C. sakazakii* (Figure 9). Hartman *et al*. [30] demonstrated that *C. sakazakii* flagellar mutants with mutations in *fliD* and *flgJ* had a reduced ability to colonize. Similarly, Grant *et al*. [31] observed that *C. jejuni* mutants in *motA* demonstrated a reduced ability to adhere to and invade INT-407 cells.

Macrophage uptake of a pathogen is an innate mechanism that helps the body to ward off the invading bacterium. However, the ability of a pathogen to survive the hostile environment and replicate within the macrophage offers it protection from the immune system and helps in disease dissemination [7]. Notably, TC significantly decreased *C. sakazakii* survival and replication in macrophages (Figure 4). Pathogens that gain access to polymorphonucelar cells and macrophages are exposed to reactive oxygen species (ROS) that function to kill bacteria. As a defense mechanism, bacteria upregulate the expression of *sod*, producing the enzyme, superoxide dismutase, to neutralize ROS. The downregulating effect of TC on *C. sakazakii sod* expression could be attributed to its reduced survival and replication within macrophages.

Endotoxin (LPS) is an important component of the outer membrane of Gram-negative bacteria that induces potent pathophysiological effects in the host [32]. As LPS is an outer membrane-associated virulence factor in *C. sakazakii*, it is proposed that the pathogen can interact with enterocytes through LPS-mediated binding to Toll-like receptors (TLR4) in the host [9]. Moreover, Deitch *et al*. [33] demonstrated that LPS facilitates bacterial attachment to Caco-2 cells and increases bacterial translocation. Our results indicated that TC reduced endotoxin production in *C. sakazakii* (Figure 8), which could be attributed to a downregulation in the expression of *lpx* and *wzx* genes (Figure 9), which are essential for the synthesis of lipid A and O antigen in Gram-negative bacteria [34].

Fluorescence microscopy of rat intestinal epithelial cells infected with *C. sakazakii* revealed a high population of dead and dying cells (Figure 5A), as compared to healthier intestinal cells in samples infected with TC-treated *C. sakazakii* (Figure 5B). *C. sakazakii* brings about cell death by inducing iNOS expression and NO production in the intestinal cells, as documented in infants with NEC and inflammatory bowel disease [17]. Quantification of iNOS expression and NO production in rat intestinal cells indicated that TC considerably down-regulated iNOS expression and reduced NO formation, compared to that in *C. sakazakii*-treated cells. Mittal *et al*. [35] observed that inhibition of iNOS expression controls pathogen load and brain damage in experimental neonatal *E. coli* meningitis; the authors proposed that prevention of NO synthesis could be a therapeutic strategy to treat neonatal bacterial meningitis. The ability of TC to bring about a reduction in NO production to a level comparable in healthy cells is suggestive of its potential application to control *C. sakazakii* infections.

## 3. Experimental Section

### 3.1. Bacterial Strains and Growth Conditions

*C. sakazakii* ATCC 29544, CS ATCC 29004 and CS 415 (meningitis isolate) were used for the study. *C. sakazaki* 415 was obtained from the Centers for Disease Control and Prevention (Atlanta, GA, USA). All bacteriological media used in the study were procured from Difco (Difco Becton, Sparks, MD, USA). Each *C. sakazakii* strain was cultured separately in 10 mL of tryptic soy broth (TSB) at 37 °C for 20 h with agitation (100 rpm). After three successive transfers, each strain of the pathogen was cultured separately in TSB at 37 °C for 20 h. Following incubation, the cultures were sedimented by centrifugation (8000× *g* for 10 min) at 4 °C. The pellet was washed twice, and resuspended in 10 mL of sterile phosphate buffered saline (PBS, pH 7.0), and serial ten-fold dilutions were cultured on duplicate tryptic soy agar (TSA), followed by incubation at 37 °C for 24 h.

### 3.2. Determination of Sub-Inhibitory Concentrations of TC

The SICs of TC were determined as we previously described [14,36]. Sterile 24-well polystyrene tissue culture plates (Costar, Corning Incorporated, Corning, NY, USA) containing TSB or tissue culture media were inoculated with ~6.0 log CFU of *C. sakazakii*, followed by the addition of 1 to 10 μL of TC (Sigma chemical Co., St. Louis, MO, USA) with an increment of 0.5 μL. The plates were incubated at 37 °C for 24 h, and bacterial growth was determined by plating on TSA plates. The three highest concentrations of TC that did not inhibit bacterial growth after 24 h of incubation were selected as sub-inhibitory concentrations for this study. Duplicate wells were included for each TC concentration and the experiment was repeated three times.

### 3.3. Motility Assay

The effect of TC on *C. sakazakii* motility was determined according to a previously published method [37]. Petriplates containing 20 mL of LB broth + 0.3% agar at 45 °C were kept still for 15–20 min. Ten μL of *C. sakazakii* culture (~6.0 log CFU) grown without or with the SICs of TC (560 or 750 μM TC) was inoculated at the center, and the plates were kept still for one hour at room temperature, followed by incubation upside down at 37 °C for 7 h. After incubation, the diameter of the motility halo (zone) was measured.

### 3.4. Cell Culture

Human embryonic intestinal cells (INT-407, ATCC, Manassas, VA, USA) were maintained in Basal Medium Eagle (Gibco, Invitrogen, Carlsbard, CA, USA) containing 10% fetal bovine serum (FBS, Invitrogen). Human enterocyte-like Caco-2 cells (ATCC HTB-27; ATCC) were maintained in MEM (Gibco, Invitrogen) containing 20% fetal bovine serum (FBS, Invitrogen). Rat intestinal epithelial cells (IEC-6, ATCC) were propagated in Dulbecco’s modified Eagle medium supplemented with 10% FBS and 1 U/mL insulin (Sigma-Aldrich, St. Louis, MO, USA). Rat intestinal cell lines were used in this study as they are an established model for study of *C. sakazakii* enterocolitis [16]. Human brain microvascular endothelial cells (BMEC) were maintained in RPMI 1640 containing 10% fetal calf serum (FCS), 10% NuSerum (Becton Dickinson, Bedford, MA, USA), modified Eagle’s medium nonessential amino acids, l-glutamine and sodium pyruvate (Invitrogen), as previously described [11].

### 3.5. Effect of SICs of TC on Binding and Internalization of C. sakazakii in Host Cells

The effect of TC on attachment and invasion of *C. sakazakii* in host cells was investigated using INT 407, Caco-2, IEC-6 and BMEC cells, according to a previously published protocol [10,16]. The cells were seeded in 24-well tissue culture plates at ~10^5^ cells per well in whole media at 37 °C in a humidified, 5% CO_2_ incubator for 18 h. *C. sakazakii* was grown to midlog phase without or with SICs of TC (325, 560 and 750 μM), centrifuged and resuspended in whole media. The cells were rinsed with minimal media and inoculated with ~6.0 log CFU (10 MOI) of *C. sakazakii* suspension. The tissue culture trays were centrifuged at 600× *g* for 5 min, and incubated at 37 °C in a humidified, 5% CO_2_ incubator. For the binding assay, the infected monolayers were rinsed three times in PBS after 1 h of incubation, and the cells were lysed with 0.1% Triton X-100. The number of viable adherent *C. sakazakii* was determined by serial dilution and plating on TSA plates. For the internalization assay, the monolayers were incubated for 1 h following infection, rinsed three times in minimal media and incubated for another 2 h in whole media-1% FBS containing gentamicin (100 g/mL) to kill the extracellular bacteria. The number of internalized bacteria was determined as described in the binding assay. The assays on each cell type were run in triplicate and replicated three times.

### 3.6. Macrophage Cultivation and Invasion Assay

Human macrophages U937 (ATCC CRL-1593.2) were maintained in RPMI 1640 medium with 10% FBS. Cells were activated and plated, as described by Townsend *et al*. [38]. Briefly, 24 h prior to infection, the cells were treated with 0.1 μg of phorbol 12-myristate 13-acetate (PMA; Sigma-Aldrich)/mL, seeded in 24-well tissue culture plates, and incubated at 37 °C under 5% CO_2_ to attach and become activated. Cells were gently washed with RPMI to remove residual PMA, and fresh medium was added prior to infection. *C. sakazakii*, grown to midlog phase in absence or presence of SICs of TC (325, 560 and 750 μM), was centrifuged and resuspended in whole media. The macrophages were infected with *C. sakazakii* at a MOI of 10 and incubated for 45 min at 37 °C under 5% CO_2_. After incubation, the macrophages were resuspended in whole medium supplemented with 100 μg of gentamicin/mL and were incubated for an additional 45 min at 37 °C under 5% CO_2_. Macrophages were then washed twice, lysed with 0.5% Triton X, serially diluted, and plated to determine the number of intracellular *C. sakazakii* at various time intervals. The results are presented as the percentage of the inoculum that was intracellular. For extended assays (intracellular replication assays), the cells were replenished with fresh medium containing 10 μg of gentamicin/mL. For each indicated time point, results are presented as percent survival of the initial intracellular population recovered at time zero. All assays were performed in triplicate at least three times.

### 3.7. Evaluation of Cell Death in IEC-6 Cells Infected with C. sakazakii

IEC-6 cells were grown to 90% confluency in 8-well chamber slides. *C. sakazakii* grown to midlog phase with TC (0 and 750 μM) was centrifuged and resuspended in whole cell media. Confluent IEC monolayers were infected with 100 MOI of *C. sakazakii* and incubated for 6 h at 37 °C under 5% CO_2_. Following incubation, the cells were washed and stained with DAPI (4-,6-diamidino-2-phenylindole) to visualize cell death using a fluorescence microscope [17].

### 3.8. Transfection of IEC-6 Cells with siRNA and Determination of Nitric Oxide (NO) Production

Transfection experiments with small interfering RNAs were performed to evaluate a potential role for nitric oxide synthase in the development of enterocolitis in *C. sakazakii* infections [35]. IEC-6 cells were grown to 50% confluency in 6-well tissue culture plates and transfected with siRNA for inducible nitric oxide synthase gene (iNOS) using lipofectamine LTX, according to the manufacturer’s instructions (Invitrogen). *C. sakazakii* was grown to midlog phase without and with TC (750 μM), centrifuged and washed with sterile PBS. Following transfection, *C. sakazakii* was added to each well at a MOI of 100 and incubated for 4 h. The supernatants were collected, cleared by centrifugation and used to estimate NO production using Griess reagent system [16], according to the manufacturer’s instructions (Promega, Madison, WI, USA). Additionally, total RNA was extracted from control (uninfected), *C. sakazakii*-infected and TC-treated -IEC-6 cells using RNeasy mini kit (Qiagen). Real-time qPCR for iNOS expression was performed using the following primers: FP (5′-TGGTGAAAGCGGTGTTCTTTG-3′) and RP (5′-ACGCGGGAAGCCATGA-3′). Ribosomal protein S-17 (RPS-17) was used as the internal control [16].

### 3.9. C. sakazakii Endotoxin Assay

Endotoxin production by *C. sakazakii* was determined using a ToxinSensor Chromogenic LAL Endotoxin Assay Kit (GenScript, Piscataway, NJ, USA). The assay was performed as per manufacturer’s instructions. *C. sakazakii* grown in TSB to midlog phase in the absence and presence of SICs of TC (560 and 750 μM) was analyzed for endotoxin production. Samples were analyzed in a 96-well format in a Bio-Rad microplate reader (Bio-Rad, Hercules, CA, USA).

### 3.10. Quantification of C. sakazakii Virulence Gene Expression Using RT-qPCR

The effect of TC on expression of *C. sakazakii* virulence genes, *fliD*, *flhD*, *motA*, *motB*, *flgJ*, *ompA*, *ompX*, *uvrY*, *lpx*, *wzx* and *sod* was investigated using real-time quantitative polymerase chain reaction (RT-qPCR, [39]). These candidate genes were identified from available literature. Each *C. sakazakii* strain was grown separately with the SICs of TC (0, 560 and 750 μM) at 37 °C in TSB to mid-log phase and total RNA was extracted using RNeasy RNA isolation kit (Qiagen, Valencia, CA, USA). Complementary DNA (cDNA) synthesis from 1 μg of RNA was performed using the Superscript II Reverse transcriptase kit (Invitrogen, Carlsbad, CA, USA). The cDNA synthesized was used as the template for RT-qPCR. Relative gene expression was assayed using the StepOne Plus Real Time PCR System (Applied Biosystems, Foster City, CA, USA). Taqman primers and probes were designed using Primer Express 3.0 from Applied Biosystems. The sequences of the primers and probes used in this study are summarized in Table 1. The probes were labeled with the reporter dye 6-carboxyfluorescein (6′-FAM) at the 5′ end and with the quencher dye NFQ-MGB at the 3′ end. Thermal cycling conditions for the quantitative PCR were as follows: 2 min at 50 °C, 10 min at 95 °C followed by 40 repeats of 15 s at 95 °C, and 1 min at 60 °C. Data were collected during each annealing phase. The data were normalized to the endogenous control (16s RNA) and the level of candidate gene expression between TC-treated and untreated samples was compared to study relative gene expression, and the effect of TC on tested genes.

### 3.11. Statistical Analysis

For each treatment and control, the data from the independent replicate trials were pooled, and analyzed using the proc mixed sub-routine of the statistical analysis software (SAS ver 9.3, SAS Institute, Cary, NC, USA). Least significant difference test was used to determine significant differences (*p* < 0.05) in assayed parameters due to treatment concentrations. Data comparisons for the gene expression study were made using Student’s *t*-test. Differences were considered significant at *p* ≤0.05.

## 4. Conclusions

In conclusion, we herein demonstrate the efficacy of SICs of TC, a natural food-grade molecule, in attenuating major virulence factors in *C. sakazakii*, highlighting its potential use in preventing and/or controlling the infection. Currently experiments screening an array of plant molecules for similar effects are underway in our laboratory. Our future studies will validate the efficacy of these plant molecules for controlling *C. sakazakii* infection in animal models.

## Figures and Tables

**Table 1.** Primers and TaqMan probes used in this study. Taqman primers and probes were designed using Primer Express 3.0 from Applied Biosystems. The probes were labeled with the reporter dye 6-carboxyfluorescein (6′-FAM) at the 5′ end and with the quencher dye NFQ-MGB at the 3′ end.

Primer	Sequence (5′ to 3′)	Probe (5′ to 3′)	Target
16F	CCAGGGCTACACACGTGCTA	AATGGCGCATACAAA	ESA_04030
16R	TCTCGCGAGGTCGCTTCT		
LF	GCACGACACTTTCCGTAAACTG	ATCAGCAGATCCGC	*lpxB*
LR	CGCCTGTTCATCGGCATT		
OAF	GGCCGCATGCCGTATAAA		
OAR	GCTGTACGCCCTGAGCTTTG	CACTGTAAACGGCGCTT	*ompA*
OXF	GTCTTTCAGCACTGGCTTGTGT	CTGGCCGTTTCCGCAG	*ompX*
OXR	GGTGCCAGCAACAGCAGAA		
Fl1F	CGATGTTTCGCCTGGGAAT	AGCGAAGAGATGGC	*flhD*
Fl1R	AGAGTCAGGTCGCCCAGTGT		
Fl2F	AAAACCGCAACATGGAATTCA	CCTCGGTCAGCAGCA	*fliD*
Fl2R	CCGCAAACGCGGTATTG		
Fl3F	GACGGCGGGCAAAGG	TTAGGCCTCGCTGACATG	*flgJ*
Fl3R	GCCGCCCATCTGTTTGAC		
M1F	GGTGTGGGTGCGTTTATCGT	CAACGGGAAAGCC	*motA*
M1R	GCCTTCAGCGTGCCTTTG		
M2F	ACGGCTCGTGGAAAATCG	TTACGCCGACTTTATG	*motB*
M2R	CCAGGAAGAAGGCCATCATG		
SF	CGAATCTGCCGGTTGAAGA		
SR	CTTGTCCGCCGGAACCT	CTGATCACCAAACTGGAT	*Sod*
UF	GCGAGGACGCCATCAAAT	TGTCGCATTCACCC	*uvrY*
UR	ATCCATCAGCACCACATCCA	ATTGCTGGGCTTAATG	*wzx*
WF	TGCTTGGGCAGGTACAAAGTG		
WR	CCCTACGGGTGCAGTCACA		
